# Predicting Difficult Tracheal Intubation in Head and Neck Cancer Patients with Osteoradionecrosis: Development of the ORN-Difficult-Airway-Score

**DOI:** 10.3390/medsci14010059

**Published:** 2026-01-27

**Authors:** Davut Deniz Uzun, Tobias Gruebl, Moritz Bleymehl, Oliver Ristow, Fabian Weykamp, Thomas Held, Stefan Mohr, Felix C. F. Schmitt, Markus A. Weigand, Juergen Debus, Kristin Uzun-Lang

**Affiliations:** 1 Department of Anesthesiology, Medical Faculty Heidelberg, University Heidelberg, 69120 Heidelberg, Germany; stefan.mohr@med.uni-heidelberg.de (S.M.); felix.schmitt@uk-gm.de (F.C.F.S.); markus.weigand@med.uni-heidelberg.de (M.A.W.); 2 Department of Anesthesiology and Intensive Care Medicine, Faculty of Medicine, Philipps University of Marburg, 35043 Marburg, Germany; tobias.gruebl@uni-marburg.de; 3 Department of Anaesthesiology, Intensive Care Medicine, Emergency Medicine and Pain Therapy, Bundeswehr Central Hospital, 56072 Koblenz, Germany; 4 Department of Oral and Cranio-Maxillofacial Surgery, Medical Faculty Heidelberg, University Heidelberg, 69120 Heidelberg, Germany; moritz.bleymehl@med.uni-heidelberg.de (M.B.); oliver.ristow@icloud.com (O.R.); 5 N2|MKG Nürnberg-Noerdlingen, 90443 Nuernberg, Germany; 6 Department of Radiation Oncology, Medical Faculty Heidelberg, University Heidelberg, 69120 Heidelberg, Germany; fabian.weykamp@med.uni-heidelberg.de (F.W.); thomas.held@med.uni-heidelberg.de (T.H.); juergen.debus@med.uni-heidelberg.de (J.D.); kristin.lang@med.uni-heidelberg.de (K.U.-L.); 7 Heidelberg Institute of Radiation Oncology (HIRO), 69120 Heidelberg, Germany; 8 Heidelberg Ion-Beam Therapy Center (HIT), Department of Radiation Oncology, Medical Faculty Heidelberg, University Heidelberg, 69120 Heidelberg, Germany; 9 Clinical Cooperation Unit Radiation Oncology, German Cancer Research Center (DKFZ), 69120 Heidelberg, Germany; 10 National Center for Tumor Diseases (NCT), 69120 Heidelberg, Germany; 11 German Cancer Consortium (DKTK), Partner Site Heidelberg, 69120 Heidelberg, Germany

**Keywords:** osteoradionecrosis, head and neck cancer, radiotherapy, difficult airway, videolaryngoscopy, fiberoptic intubation, airway assessment, prediction score

## Abstract

Background: Osteoradionecrosis (ORN) following head and neck radiotherapy has been demonstrated to induce structural and functional alterations of the upper airway, with the potential to complicate the process of tracheal intubation. Despite its clinical relevance, there is a paucity of systematic evidence on airway characteristics in ORN and reliable predictors of difficult tracheal intubation. This study compares preoperative airway parameters and tracheal intubation outcomes in irradiated patients with and without ORN and introduces a novel preoperative ORN-Difficult-Airway Score for risk stratification. Methods: In this retrospective cohort study, airway assessments, tracheal intubation methods, and perioperative visualization parameters were evaluated in 105 patients following head and neck radiotherapy. Group differences between non-ORN and ORN were analyzed using chi-square tests. A preoperative ORN-Difficult-Airway Score was constructed using exclusively bedside parameters, based on statistically and clinically relevant predictors. Results: Patients with ORN showed significantly restricted mouth opening (*p* < 0.001), higher Mallampati classes, particularly Mallampati IV, and a greater need for fiberoptic tracheal intubation (*p* < 0.01). Direct laryngoscopy (DL) was significantly less feasible in ORN, while hyperangulated videolaryngoscopy (VL) yielded consistently positive visualization (first-pass success (FPS) 100% in both groups). Under DL, FPS was lower in ORN (54.2% vs. 79.5%), resulting in an odds ratio of 0.305. Based on observed predictors, ORN status, mouth opening <3 cm, Mallampati class, restricted neck reclination, and history of difficult intubation, a preoperative ORN-Difficult-Airway Score was developed. Conclusions: ORN has been associated with distinct alterations in airway anatomy and visualization, resulting in increased tracheal intubation complexity after head and neck radiotherapy. The proposed ORN-Difficult-Airway Score presents a clinically practical, bedside-applicable approach to stratifying the risk of tracheal intubation in this population. Prior to clinical implementation, prospective validation in larger cohorts is warranted.

## 1. Introduction

Osteoradionecrosis (ORN) is a grave late complication that manifests following radiation therapy (RT) in the head and neck region. The incidence of ORN varies depending on the definition, radiation dose, and quality of follow-up care. However, it is reported in the literature as 4–15%, with the lower jaw being affected significantly more often than the upper jaw due to its lower vascular supply [1,2,3,4]. Pathophysiologically, ORN is based on a radiation-induced combination of hypovascularity, hypocellularity, and hypoxia, which leads to chronic wound healing disorders and bone necrosis [5,6]. ORN is defined as exposed, irradiated bone that does not heal over a period of 3 months without evidence of persistent or recurrent tumor [7,8]. Radiological evidence of bone necrosis within the target volume is also important for diagnosis and classification [9]. In addition to local complications, such as pain, infections, and pathological fractures, ORN has implications for advanced airway management [10]. Patients who have previously undergone RT in the head and neck area often exhibit several adverse effects, including trismus, soft tissue fibrosis, and altered topography of the upper respiratory tract [11,12,13]. Consequently, procedures such as mask ventilation, laryngoscopy, and tracheal intubation become significantly more challenging [13]. The guideline of the German Society for Anesthesiology and Intensive Care Medicine (DGAI) on “Securing the airway in adults” explicitly points out that previous RT in the head and neck area should be considered a relevant risk factor for a difficult airway [14]. The scientific literature on the direct impact of ORN on advanced airway management is currently limited [15,16]. However, the extant data demonstrate a consistent increase in complication rates and the necessity of specific preparation and technique. In light of these risks, a meticulous preoperative airway assessment is imperative, encompassing fiberoptic tracheal intubation (FOI) when indicated. Anesthetic strategies frequently necessitate the early implementation of videolaryngoscopy (VL), meticulous preparation for awake FOI, and extubation planning, in addition to postoperative monitoring [17]. A comprehensive understanding of radiation-induced anatomical and functional alterations is imperative for anesthesiologists when managing patients who have previously undergone head-and-neck irradiation. Such knowledge facilitates the early recognition of airway-related risks, supports proactive planning of advanced airway strategies, and ultimately contributes to reducing perioperative morbidity and enhancing patient safety [10].

A significant gap in the extant literature pertains to the paucity of validated tools to systematically predict airway difficulty, with particular reference to patients with ORN. In view of the increasing number of cancer survivors undergoing RT and the growing complexity of their reconstructive and surgical interventions, there is an urgent need for a structured, evidence-based risk stratification instrument. The ORN-Difficult-Airway Score, which was introduced in this study, represents a novel attempt to address this gap by identifying key predictors of difficult airway management in this unique patient population.

The objective of this study was twofold: first, to evaluate the influence of ORN on advanced airway management, including first-pass success (FPS) and required airway interventions, and second, to develop and propose the ORN-Difficult-Airway Score as a preliminary risk assessment tool. With external validation in larger multicenter cohorts, this score has the potential to support anesthesiologists in preoperative decision-making and to enhance perioperative patient safety.

## 2. Materials and Methods

### 2.1. Study Design and Patient Selection

The present study was conducted as a retrospective cohort analysis, including all patients diagnosed and treated within the defined time period from January 2015 to January 2025. A comprehensive data set encompassing clinical, tumor-related, airway-related, and treatment-related variables was meticulously extracted from the electronic medical records. Patients were grouped according to the presence or absence of ORN, as defined by clinical and radiological criteria. This retrospective study was conducted in accordance with the relevant institutional guidelines and in compliance with the Declaration of Helsinki of 1975, as revised in 2013. The study was approved by the local ethics committee of the medical faculty at the University of Heidelberg (reference number S-421-2015).

### 2.2. Variables and Definitions

The airway assessment parameters included the Mallampati score (grades I–IV), mouth opening (≥3 cm vs. <3 cm), neck reclination (good vs. restricted), mask ventilation (graded as easy or difficult), tracheal intubation methods (direct laryngoscopy (DL), videolaryngoscopy (VL), fiberoptic tracheal intubation (FOI), or tracheostomy), and first-pass intubation success (FPS). Cardiovascular risk factors (CVRF) were incorporated into the study as binary variables. Tumor staging (T-stage, N-stage) and American Society of Anesthesiologists (ASA) classification were obtained from clinical documentation. The outcome parameters were defined similarly to those in the HAPA registry [18]. The clinical target volume (CTV) is defined as the volume encompassing the gross tumor and adjacent tissues at risk of microscopic disease. This volume is extracted from radiotherapy planning data.

### 2.3. Statistical Analysis

Descriptive statistics were used to summarize all variables. Categorical data were reported as absolute frequencies and percentages in each group. Continuous variables (e.g., age) were handled descriptively but not included in categorical comparative testing.

### 2.4. Group Comparisons

The differences between non-ORN and ORN were assessed using Pearson’s chi-square test for categorical variables. The following testing approach was applied:

- ASA classification;
- Tumor T-stage and N-stage;
- Radiation dose groups;
- Cardiovascular risk factors;
- Mallampati score;
- Mouth opening;
- Neck reclination;
- Mask ventilation;
- Intubation method distribution;
- First-pass success.

In instances where a contingency table exhibited one or more zero cells, a Haldane–Anscombe correction was implemented. This correction involved the addition of 0.5 to each cell, with the objective of enhancing test stability.

### 2.5. Stratified Airway Analyses

For airway management-related outcomes, additional stratified analyses were performed. FPS was evaluated separately for DL and VL after exclusion of FOI and tracheostomy cases to ensure methodological consistency.

### 2.6. Odds Ratios and Statistical Thresholds

For comparisons resulting in 2 × 2 contingency tables, odds ratios (OR) were calculated. In instances where necessary, a 0.5 continuity correction was applied. A *p*-value of <0.05 was considered statistically significant. All statistical tests were two-sided. Analyses were performed using IBM SPSS Statistics (Version 30).

### 2.7. ORN-Difficult-Airway Score

The ORN-Difficult-Airway Score was constructed as an exploratory, clinically oriented risk stratification tool using exclusively bedside airway parameters. Variable selection was based on a combination of statistical associations observed in the present dataset and established relevance in the difficult airway literature. Tumor-related variables (tumor localization, T-stage, N-stage), surgical history, and radiotherapy-related parameters were systematically extracted from clinical and radiotherapy records. These variables were not included in the score construction, as the primary aim was to develop a bedside-applicable tool based exclusively on parameters available during routine preoperative airway assessment. Score weighting was intentionally not derived from regression coefficients alone, given the limited sample size and the risk of model overfitting. Instead, a hybrid clinical-statistical approach was applied. Parameters demonstrating both a strong association with ORN and a well-documented impact on laryngoscopic feasibility were weighted more heavily.

Reduced mouth opening (<3 cm) was assigned 2 points, as it showed the most pronounced difference between groups and represents a critical limiting factor for both direct and VL. Mallampati classification was graded incrementally (Class III = 1 point; Class IV = 2 points) to reflect increasing oropharyngeal obstruction. ORN status, restricted neck reclination, and a documented history of difficult tracheal intubation were each assigned 1 point, reflecting their consistent but comparatively lower individual effect sizes and their established clinical relevance. This pragmatic weighting strategy was chosen to enhance bedside usability and clinical interpretability. The resulting score is therefore intended as an exploratory risk stratification instrument, suitable for guiding preoperative airway planning rather than providing definitive probability estimates. Definition of the proposed exploratory ORN-Difficult-Airway Score shown in Table 1. The interpretation and risk stratification of the Score are shown in Table 2.

## 3. Results

### 3.1. Patient Characteristics

A total of 105 patients were included in the analysis, of whom 53 were classified as non-ORN and 52 as ORN. Patients in both groups were predominantly male (non-ORN = 84.9%; ORN = 78.8%). The median age in the non-ORN group was 73.2 years and 74.8 years in the ORN group. Table 3 shows the detailed baseline characteristics.

### 3.2. Airway Management Methods

Advanced airway management techniques were evaluated and grouped into four mutually exclusive categories: DL, VL, FOI, and tracheostomy. The distribution of these methods differed between the two groups. DL was performed in 39 of 53 patients (73.6%) in the non-ORN group and in 24 of 52 patients (46.2%) in the ORN group (*p* = 0.0076). VL was used in 8 patients (15.1%) in the non-ORN group and in 11 patients (21.2%) in the ORN group (*p* = 0.5803). FOI was documented in 5 patients (9.4%) in the non-ORN group and in 15 patients (28.8%) in the ORN group (*p* = 0.0224). Tracheostomy was performed in 1 patient (1.9%) in the non-ORN group and in 2 patients (3.8%) in the ORN group (*p* = 0.9866). Of the variables examined, three parameters exhibited significant differences between patients with and without ORN (Table 3). Patients who developed ORN received a higher mean total dose (66.9 Gy vs. 65.4 Gy; *p* = 0.001). Furthermore, a substantial increase in CTV was observed in the boost plan (185 mL vs. 109 mL; *p* = 0.004), suggesting a more extensive or intensively treated tumor region. Furthermore, the Mallampati score was higher in the ORN group (3.17 vs. 2.64; *p* = 0.003), suggesting anatomical differences in the oropharynx. The study revealed no statistically significant differences between the groups with respect to age, gender, CTV in the basic plan, maximum dose to the mandible (Dmax), tumor T stage, nodal status, and ASA classification. The findings indicate that both dosimetric factors (total dose, boost volume) and anatomical parameters (Mallampati score) play a significant role in the management of difficult airways. Analysis of clinical patient characteristics (ASA classification, tumor stages, nodal status), the radiation dose administered, and relevant cardiovascular risk factors (CVRF) revealed no statistically significant differences between patients without ORN and those who developed ORN (Table 3).

### 3.3. ASA Classification

The distribution of ASA classes was similar between the two groups. The non-ORN group consisted predominantly of ASA III patients (67.9%), followed by ASA II (28.3%). The pattern was similar in the ORN group (ASA III: 61.5%, ASA II: 38.5%). The difference was not statistically significant (*p* = 0.401).

#### Tumor Localisation, T Stage, and N Status

Oral cavity cancers were more frequently observed in the ORN group (86.5%) compared with the non-ORN group (58.5%), whereas laryngeal cancers were more common in the non-ORN group (41.5% vs. 13.5%). The tumor stage showed a slight shift toward higher T stages in the ORN group: While T1 and T2 dominated in the non-ORN group (35.8% and 37.7%, respectively), the ORN group showed a higher proportion of T2 (51.9%) and T4 tumors (19.2%) (Table 3). The tumor stage showed a slight shift toward higher T stages in the ORN group: While T1 and T2 dominated in the non-ORN group (35.8% and 37.7%, respectively), the ORN group showed a higher proportion of T2 (51.9%) and T4 tumors (19.2%). Despite these trends, the comparison did not reach statistical significance (*p* = 0.109). Nodal involvement was similarly distributed in both groups. In the non-ORN group, 75.5% of patients had a positive N status (N+), compared with 67.3% in the ORN group. Here, too, there was no significant difference (*p* = 0.478).

### 3.4. Neck Reclination and Mask Ventilation

In the non-ORN group, good neck reclination was documented in 41 of 53 patients (77.4%), while 12 patients (22.6%) had limited reclination. In the ORN group, favorable reclination was observed in 38 of 52 patients (73.1%), while 13 patients (25.0%) exhibited limited reclination. A subsequent statistical comparison of the distributions revealed no significant disparities between the groups (*p* = 0.564). Following the exclusion of cases that could not be assessed, as during fiberoptic tracheal intubation, ventilation is not used, an analysis of mask ventilation was performed in 46 patients in the non-ORN group and 35 patients in the ORN group. Easy mask ventilation was documented in 29 of 46 patients (63.0%) in the non-ORN group and in 22 of 35 patients (62.9%) in the ORN group. Difficult mask ventilation was diagnosed in 17 patients (37.0%) and 13 patients (37.1%), respectively. A comparison of the two groups revealed no significant difference (*p* = 1.000) (Table 4).

### 3.5. First Pass Intubation Success

**Figure 1:**
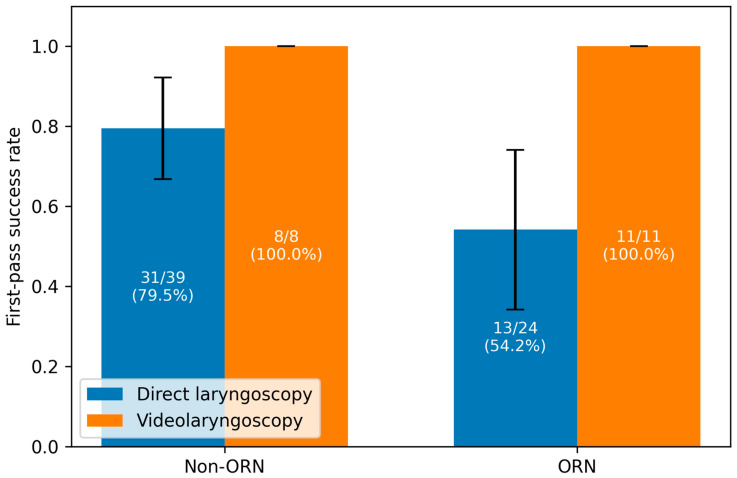
First-pass success (FPS) rates for direct laryngoscopy (DL) and videolaryngoscopy (VL) in patients with and without osteoradionecrosis (ORN). Error bars indicate 95% confidence intervals.

After excluding FOI and tracheostomies, the FPS was analyzed separately for VL and DL. Under VL, all patients in both the non-ORN group (8/8; 100%) and the ORN group (11/11; 100%) showed a positive FPS (*p* = 1000). During DL, a positive FPS was present in 31 of 39 cases (79.5%) in the non-ORN group and in 13 of 24 cases (54.2%) in the ORN group, without statistical significance (*p* = 0.065) (Figure 1, Table 4).

### 3.6. Characteristics of Radiotherapy Treatment

Patients diagnosed with ORN demonstrated elevated levels of radiation exposure across multiple dosimetric parameters. Despite the mean clinical target volume (CTV) not demonstrating a significant difference between the groups (657.98 mL vs. 795.89 mL; *p* = 0.114), the boost CTV exhibited a substantial increase in the ORN cohort (185 mL vs. 109 mL; *p* < 0.05), suggesting a more extensive or intensively irradiated tumor region. In a similar vein, the mean total radiation dose was found to be significantly higher in patients who subsequently developed ORN (66.9 Gy vs. 65.4 Gy; *p* < 0.05). Conversely, the maximum mandibular dose (Dmax) exhibited no statistically significant discrepancy between the groups (59 Gy vs. 56 Gy; *p* = 0.397).

### 3.7. ORN-Difficult-Airway Score

The discriminatory ability of the ORN-Difficult Airway Score was assessed using receiver operating characteristic (ROC) curve analysis and the corresponding area under the curve (AUC). Difficult tracheal intubation was defined as the requirement for fiberoptic tracheal intubation or tracheostomy, or a Cormack–Lehane grade ≥ III. Sensitivity, specificity, and the positive and negative predictive values (PPV and NPV) were calculated for the predefined score thresholds of ≥3 and ≥4 points. The internal validation was executed through the implementation of bootstrap resampling, encompassing 500 iterations, to derive an optimism-corrected estimate of the area under the curve (AUC).

### 3.8. Performance of the ORN-Difficult-Airway Score

**Figure 2:**
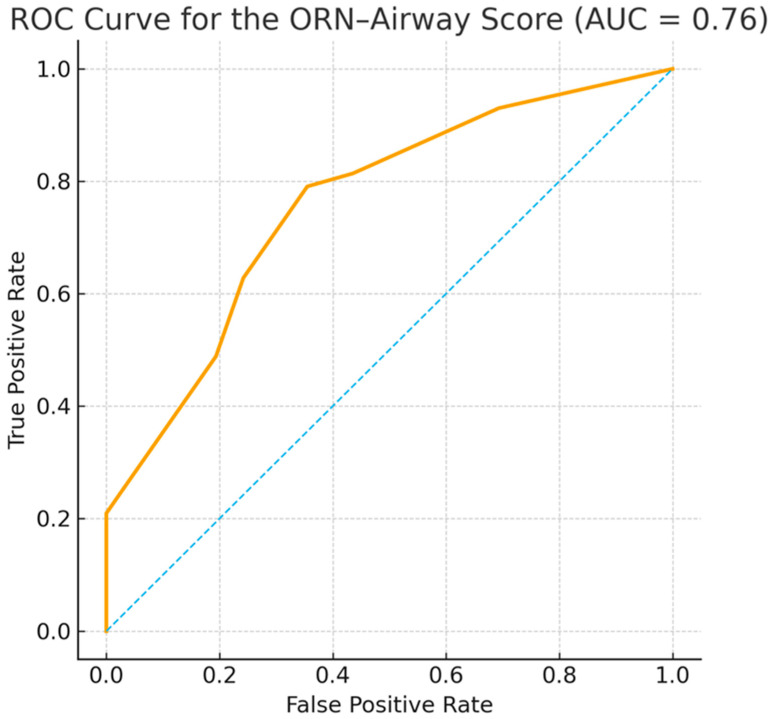
Receiver operating characteristic (ROC) Curve for the ORN-Difficult-Airway Score.

The ORN-Difficult-Airway Score’s capacity to predict difficult intubation was assessed through receiver operating characteristic (ROC) analysis, employing all patients with complete airway data (*n* = 105). Difficult intubation was defined as the need for fiberoptic tracheal intubation or tracheostomy, or a Cormack–Lehane grade ≥ III. The Score demonstrated an area under the curve (AUC) of 0.76, indicating acceptable discriminatory performance in distinguishing between difficult and non-difficult intubations (Figure 2). The internal validation process, employing bootstrap resampling with 500 iterations, yielded a mean optimism-corrected AUC of 0.76 (SD 0.05), indicating stable internal performance and minimal overfitting. When a predefined cutoff of ≥4 points is applied to define a high-risk airway, the score achieves a sensitivity of 62.8%, a specificity of 75.8%, a positive predictive value (PPV) of 64.3%, and a negative predictive value (NPV) of 74.6%. A lower cutoff of ≥3 points increased the sensitivity to 79.1% with a specificity of 64.5% (PPV 60.7%, NPV 81.6%). The findings, when considered collectively, suggest that the ORN-Difficult-Airway Score offers clinically meaningful discrimination of airway difficulty in patients with and without ORN following head and neck radiotherapy. In order to confirm the external generalizability of the findings, prospective validation in larger cohorts is required.

### 3.9. The Utilization of the Score in the Group Under Study

The ORN-Difficult-Airway Score was calculated for all 105 patients based on four preoperative airway parameters (ORN status, mouth opening, Mallampati classification, and neck reclination), resulting in total scores ranging from 0 to 7 points. Patients were categorized into three predefined risk strata: low risk (0–1 points), intermediate risk (2–3 points), and high risk (≥4 points). In the overall cohort, 43 patients (41.0%) were classified as low risk, 20 patients (19.0%) as intermediate risk, and 42 patients (40.0%) as high risk. Stratification by ORN status revealed a pronounced shift toward higher risk profiles among patients with radiotherapy-associated ORN. In the non-ORN group (*n* = 53), 33 patients were categorized as low risk, 10 as intermediate risk, and 10 as high risk. In contrast, the ORN group (*n* = 52) included only 10 low-risk and 10 intermediate-risk patients, while 32 patients fell within the high-risk category. Risk categories also correlated with the observed difficulty of airway management. Among patients without difficult intubation (*n* = 62), 35 were classified as low risk, 12 as intermediate risk, and 15 as high risk. Conversely, in the subgroup with difficult tracheal intubation (*n* = 43), only 8 patients were classified as low risk and 8 as intermediate risk, whereas 27 patients (63%) were assigned to the high-risk category. These distributions correspond closely with the discriminatory performance of the ORN-Difficult-Airway Score in ROC analysis.

## 4. Discussion

The present study demonstrates that ORN following head-and-neck RT is associated with distinct, clinically relevant alterations of airway anatomy that significantly affect the feasibility of tracheal intubation. In light of the aforementioned findings, we propose the ORN-Difficult-Airway Score as a pragmatic, bedside-based tool for preoperative risk stratification. The central clinical message of this work is not the superiority of a specific device, but rather the necessity of structured airway risk recognition and proactive planning in this high-risk population. This topic is of significant clinical importance because the absence of a comprehensive preoperative anesthesiologic evaluation can result in potentially life-threatening complications, including “cannot intubate, cannot oxygenation” (CICO) and, in extreme cases, death [19]. The analysis revealed significant differences between the two groups, particularly with regard to functional airway parameters and tracheal intubation techniques. These results may indicate radiation-related anatomical and functional changes and underscore the clinical importance of a structured approach to assessing airways in this patient population.

The ORN-Difficult-Airway score incorporates only bedside parameters available during routine preoperative evaluation. The ensuing clinical variables exhibited substantial or clinically meaningful associations with ORN and with markers of difficult airway management in the present analyses: The analysis revealed a robust correlation between reduced mouth opening and ORN, thereby establishing this parameter as a reliable predictor of challenging laryngoscopy in the broader population. This finding aligns with previous systematic reviews demonstrating that radiotherapy for head and neck cancer frequently results in trismus, i.e., limitation of maximal mouth opening, which increases over time and correlates with dose and treated volume [20,21]. A higher Mallampati classification, specifically Mallampati IV, was observed with greater frequency in patients with ORN.

Impaired neck reclination exhibited a higher prevalence in patients with ORN, and its impact on laryngoscopic visualization is well-documented [16,22]. In clinical practice, these anatomical alterations translated into a significantly increased need for FOI and reduced feasibility of DL in patients with ORN. A documented history of difficult intubation, when available, remains a universally acknowledged risk factor and was therefore incorporated to enhance predictive accuracy [12,13,23,24,25]. To ensure clinical usefulness, the variables were weighted according to their relative effect in the dataset and their established relevance in the airway literature. In line with international difficult airway guidelines, the score is intended to support proactive perioperative planning by identifying patients who may benefit from early preparation of advanced airway strategies, including VL or FOI [26]. Laryngeal ultrasonography represents a complementary bedside tool for airway assessment in patients with anticipated difficult tracheal intubation. It enables non-invasive visualization of the laryngeal framework and surrounding soft tissues and may be particularly useful in patients with altered anatomy after head-and-neck radiotherapy. During the SARS-CoV-2 pandemic, laryngeal sonography gained attention as a rapid and safe airway assessment method, as reviewed by Cergan et al. [27]. Although not a replacement for established airway techniques, it may support preoperative airway planning in patients with restricted mouth opening or distorted airway anatomy.

Based on the identified associations between airway parameters and ORN status, a composite risk score for predicting difficult intubation in irradiated head and neck cancer patients appears conceptually feasible. Particularly, reduced mouth opening, higher Mallampati classes, and impaired neck reclination emerged as consistent markers of increased airway complexity. An area under the receiver operating characteristic curve of 0.76 indicates the model’s good discriminatory performance. This value shows that the score can distinguish between outcome groups with satisfactory accuracy, which is considered acceptable and meaningful in predictive modeling.

FOI was used significantly more often in patients with ORN, whereas DL was more frequently feasible in patients without ORN. These findings support the theory that structural and functional changes associated with ORN contribute to a more complex airway. Conversely, there was no significant difference in the usage of VL between groups, which may reflect its broader role in difficult airway protocols beyond the effects of radiotherapy [14,26,28]. All VL intubations demonstrated FPS irrespective of ORN status, whereas direct laryngoscopy showed a lower FPS positivity in the ORN group, although not reaching statistical significance. These results suggest that VL may provide consistent visualization, even in anatomically altered airways, while DL may be more susceptible to radiation-induced structural alterations. The calculated odds ratio for FPS under DL further supports the idea that visualization quality is reduced in ORN patients.

It should be noted that VL is not universally applicable, particularly in cases of severely restricted mouth opening, and that intubation success remains dependent on operator experience and appropriate adjunct use. Nevertheless, the data support the theory that experience with the hyperangulated VL blade significantly influences FPS [29].

The continued use of DL in a substantial proportion of patients with ORN highlights the need for improved preoperative risk stratification and guideline-concordant airway planning. This is particularly relevant given the well-established association between repeated intubation attempts and increased complication rates [30,31]. However, VL is not without its limitations. The reduced mouth opening has already been discussed. Furthermore, in the current era of hyperangulated VL, visualization of the glottis level is almost never an issue. Rather, particular difficulties arise in placing the tracheal tube through the vocal cords. This is because the lines of sight are different compared to conventional DL, and the tube has to make two turns. Greenland et al.’s explanatory model provides a very good explanation for the two-curve theory [32]. From a clinical perspective, the ORN-Difficult-Airway Score may support anesthesiologists by the following:

(1) identifying irradiated patients at increased risk of difficult tracheal intubation;

(2) prompting early preparation of videolaryngoscopy or fiberoptic techniques;

(3) reducing unplanned escalation during airway management.

Several oncologic and treatment-related factors, including tumor localization, radiation dose, and prior surgical interventions, may influence upper airway anatomy and intubation difficulty. In the present study, these variables were systematically assessed and differed partially between patients with and without osteoradionecrosis. However, they were deliberately not incorporated into the ORN-Difficult-Airway Score. The primary objective of the score was to provide a pragmatic, bedside-based risk stratification tool applicable during routine preoperative assessment, independent of detailed oncologic or radiotherapy planning data, which are often unavailable at the time of anesthetic evaluation. Consequently, residual confounding related to tumor- and treatment-specific factors cannot be fully excluded and should be addressed in future prospective validation studies.

This study has several important limitations that should be acknowledged. First, its retrospective, single-center design inherently limits causal inference and may introduce selection and documentation bias. Although all eligible patients within the defined study period were included, unmeasured confounders cannot be fully excluded.

Second, the overall sample size was relatively small, particularly for the development of a multivariable predictive score. While this cohort represents a clinically well-defined and highly relevant patient population, limited sample size may affect model stability and generalizability. For this reason, the ORN-Difficult-Airway Score should be regarded as exploratory and hypothesis-generating, rather than as a definitive clinical prediction tool. To mitigate overfitting, internal validation was performed using bootstrap resampling, which demonstrated stable discriminatory performance; however, external validation in larger, prospective, multicenter cohorts is mandatory before clinical implementation can be recommended. Third, although relevant oncologic and treatment-related variables such as tumor localization, tumor stage, and radiation dose were collected and compared between groups, the study was not designed to fully adjust for all potential confounding factors influencing airway anatomy and intubation difficulty. Importantly, these variables were deliberately not incorporated into the score itself, as the primary aim was to develop a bedside-applicable tool based exclusively on parameters available during routine preoperative airway assessment. Fourth, calibration analysis and decision curve analysis were not performed. Given the limited sample size and retrospective nature of the dataset, such analyses may yield unstable estimates and were therefore deferred to future prospective validation studies, where clinical decision thresholds can be defined more robustly. Finally, airway management outcomes are influenced not only by anatomical factors and technique selection but also by operator experience and institutional expertise. As the present study was conducted at a tertiary referral center with substantial experience in managing irradiated head-and-neck cancer patients, the findings may not be directly transferable to settings with different levels of expertise.

## 5. Conclusions

In this retrospective cohort of irradiated head-and-neck cancer patients, ORN was associated with distinct structural and functional airway alterations that substantially influenced the feasibility of advanced airway techniques. Patients with ORN demonstrated reduced suitability for DL and a markedly increased need for FOI, thereby underscoring the clinical relevance of radiation-induced airway changes. Building on these observations, the ORN-Difficult-Airway Score was developed. This is a novel preoperative risk stratification tool derived exclusively from bedside airway parameters. The score demonstrated good discriminatory performance (AUC 0.76) and a meaningful correlation between higher risk strata and difficult tracheal intubation events, indicating its potential utility in guiding perioperative planning and the early use of advanced airway strategies such as VL or FOI techniques.

Whilst these findings emphasize the significance of structured airway assessment in patients who have previously undergone head-and-neck irradiation, it should be noted that the proposed score remains exploratory. Robust external validation in larger, prospective, multicenter cohorts is essential before clinical implementation can be recommended. Nevertheless, the present study provides a foundation for establishing evidence-based airway risk assessment in a population with increasing prevalence and high perioperative vulnerability.

## Figures and Tables

**Table 1:** Definition of the proposed exploratory ORN–Difficult-Airway Score. Maximum score is 7 points.

Variable	Clinical Definition	Points
Osteoradionecrosis (ORN)	No = 0; Yes = 1	0–1
Mouth opening	≥3 cm = 0; <3 cm = 2	0–2
Mallampati classification	Class I–II = 0	
	Class III = 1	0–2
	Class IV = 2	
Neck reclination	Normal = 0; Restricted = 1	0–1
History of a difficult airway	No = 0; Yes = 1	0–1

**Table 2:** Interpretation and risk stratification of the ORN-Difficult-Airway Score.

Total Score	Risk Category	Clinical Interpretation
0–1 Points	Low risk	Unremarkable airway; direct laryngoscopy likely feasible
2–3 Points	Intermediate risk	Selected difficulty markers present; videolaryngoscopy recommended as primary tool
≥4 points	High risk	Videolaryngoscopy or fiberoptic tracheal intubation; prepare rescue strategies

**Table 3:** Baseline patients’ characteristics.

Baseline Characteristics	Non-Osteoradionecrosis	Osteoradionecrosis	*p*-Value
Gender Female Male	n of patients, (%)	n of patients, (%)	
	8 (15.1%)	11 (21.2%)	0.580
	45 (84.9%)	41 (78.8%)	0.580
Body size (cm) Body weight (kg)	175.4	173.3	0.231
	75.4	71.7	0.176
Age	73.2	74.8	0.406
ASA classification			0.401
I (healthy)	1 (1.9%)	0 (0.0%)	
II (Mild systemic illness)	15 (28.3%)	20 (38.5%)	
III (Severe systemic illness)	36 (67.9%)	32 (61.5%)	
IV (Life-threatening systemic illness)	1 (1.9%)	0 (0.0%)	
Tumor localization			**<0.05**
Laryngeal cancer	22 (41.5%)	7 (13.5%)	
Oral cavity cancer	31 (58.5%)	45 (86.5%)	
T-stage			0.109
T1	19 (35.8%)	8 (15.4%)	
T2	20 (37.7%)	27 (51.9%)	
T3	7 (13.2%)	7 (13.2%)	
T4	7 (13.2%)	10 (19.2%)	
N-status			0.478
N0	13 (24.5%)	17 (32.7%)	
N1	40 (75.5%)	35 (67.3%)	
CTV (mL)	657.98	795.89	0.114
CTV boost (mL)	109	185	**<0.05**
Mean total dose (Gy)	65.4	66.9	**<0.05**
Dmax mandibula (Gy)	56	59	0.397
Cardiovascular risk factors			
Hypertension	20 (37.7%)	14 (26.9%)	0.329
Smoking	35 (66.0%)	31 (59.6%)	0.632
Coronary artery disease	11 (20.8%)	10 (19.2%)	1.000
Heart failure	3 (5.7%)	3 (5.8%)	1.000
Diabetes mellitus	1 (0.9%)	0 (0.0%)	1.000

**Table 4:** Anesthesia-related characteristics.

Baseline Characteristics	Non-Osteoradionecrosis	Osteoradionecrosis	*p*-Value
Mallampati score			**<0.05**
I (Soft palate, uvula, pillars visible)	5 (9.4%)	0 (0.0%)	
II (Soft palate, major part of uvula visible)	20 (37.7%)	17 (32.7%)	
III (Soft palate, base of uvula visible)	17 (32.1%)	9 (17.3%)	
IV (Only hard palate visible)	11 (20.8%)	26 (50.0%)	
Mouth opening			**<0.001**
<3 cm	38 (71.7%)	39 (75.0%)	
>3 cm	15 (28.3%)	13 (25.0%)	
Neck range of motion			0.564
Full	41 (77.4%)	38 (73.1%)	
Limited	12 (22.6%)	13 (25.0%)	
Not documented	0 (0.0%)	1 (1.9%)	
Mask ventilation			1.000
Easy	29 (63.0%)	22 (62.9%)	
Difficult	17 (37.0%)	13 (37.1%)	
Tracheal intubation technique			
Direct laryngoscopy (DL)	39 (73.6%)	24 (46.2%)	**<0.05**
Video laryngoscopy (VL)	8 (15.1%)	11 (21.2%)	0.580
Fiberoptic tracheal intubation (FOI)	5 (9.4%)	15 (28.8%)	**<0.05**
Urgent tracheostomy	1 (1.9%)	2 (3.8%)	0.986
First-Pass Intubation Success (FPS)			
Direct laryngoscopy (DL)	31 (79.5%)	13 (54.2%)	0.065
Video laryngoscopy (VL)	8 (100%)	11 (100%)	1.000

## Data Availability

The original contributions presented in this study are included in thearticle. Further inquiries can be directed to the corresponding author.

## Abbreviations

The following abbreviations are used in this manuscript:

ASA	American Society of Anesthesiologists
CICO	Cannot Intubate, Cannot Oxygenate
cm	Centimeter
DL	Direct Laryngoscopy
FOI	Fiberoptic Tracheal Intubation
Gy	Gray
Kg	Kilogram
NPV	Negative Predictive Value
ORN	Osteoradionecrosis
PPV	Positive Predictive Value
ROC	Receiver Operating Characteristic
VL	Videolaryngoscopy
